# Supplementary material for: MetaRibo-Seq measures translation in microbiomes
Source: Nat Commun. 2020 Jun 29;11:3268. doi: 10.1038/s41467-020-17081-z (PMC7324362; doi:10.1038/s41467-020-17081-z)
Supplement: Supplementary file 10 — Supplementary Data 7 [file 41467_2020_17081_MOESM10_ESM.zip › File2/Confidence_High_Taxonomy/25935_out.krona.html]

Javascript must be enabled to view this page.

members
magnitude
magnitudeUnassigned
count
unassigned
taxon
rank

25935\_out

2

2
superkingdom
2

1239
phylum
2

class
186801
2

186802
order
2

family
541000
2

1
genus
292632

2053618
species
1

SRS050941\_contig\_number\_14568

1

SRS148159\_contig\_number\_23359
species
1898205
